# CLEAR CELL SARCOMA: A CASE MIMICKING PRIMARY CUTANEOUS MALIGNANT MELANOMA

**DOI:** 10.4103/0019-5154.53193

**Published:** 2009

**Authors:** M Rodríguez-Martín, M Sáez-Rodríguez, B Esquivel, R Sánchez Gonzáalez, A Noda Cabrera, A Martín Herrera

**Affiliations:** *From the Departments of Dermatology, Hospital Universitario de Canarias, University of La Laguna, 38320 – La Laguna, Tenerife, Spain*; 1 *From the Department of Pathology, Hospital Universitario de Canarias, University of La Laguna, 38320 – La Laguna, Tenerife, Spain*

**Keywords:** *Clear cell sarcoma*, *genetic studies*, *malignant melanoma*

## Abstract

Clear cell sarcoma (CCS) is a recently described variant of sarcoma characterized by prominent clear cells showing features similar to clear cell melanoma. This neoplasm was first described by Dr. Franz M. Erzinger. Primary CCS usually arises in deeper soft tissues, in association with fascia, tendons, or aponeuroses. Characteristic translocation t(12;22) (q13;q12) has been considered pathognomonic for CCS. Prognosis is related to tumor size. An early recognition and initial radical surgery is the key to a favourable outcome. We present a patient with an unusual neoplasm that resembled malignant melanoma.

## Introduction

Clear cell sarcoma (CCS) is a rare neoplasm with a difficult clinical and histological differential diagnosis. The entity of CCS is also known as *malignant melanoma of soft parts* and it represents about 1% of soft tissue tumors. Because of the presence of melanin, pre-melanosomes, S-100 protein and the tendency for regional nodal metastases, it has been suggested that this entity may be considered as melanoma rather than soft tissue sarcoma.[1] According to some authors, *clear cell sarcoma of tendons and aponeuroses* should be the proper name for this rare tumor, to prevent confusion with CCS of the kidney and other clear and spindle-cell neoplasms.[2,3] Clinically, most cases present as a slowly progressive, painless mass on the lower limbs with a predilection for young females. The tumor increases in size followed by metastatic dissemination to lymph nodes and lungs. Malignant melanoma (MM) is the most important differential diagnosis to exclude. However, unusual histologic variants of MM may be problematic to differentiate based on routine histopathologic evaluation. Characteristic translocation t(12;22) (q13;q12) has been considered pathognomonic for CCS. This translocation has been observed in neither cutaneous or uveal MM nor malignant peripheral nerve sheath tumor (MPNSTs+).[3–6] This genetic traslocation demonstrates that CCS resembles MM but has a different pathogenesis.

An unusual clinical case is presented. We describe a male patient with a cutaneous lesion on the arm, arising in a preexisting pigmented lesion with histological features that resembled melanoma. A rare variant of sarcoma mimicking clear cell melanoma was diagnosed based on the genetic studies. To our knowledge, this is the first reported case of CCS showing intraepidermal involvement.

## Case Report

A 53-year-old caucasian male presented to our Department with a painful lesion on the right arm. He related that the pigmented lesion had remained stable for years, with no pain or increase in size. Over the past 4 months, he stated that it began to enlarge and became painful. Medical history was relevant for a pheochromocytoma treated by excisional surgery with adrenalectomy two years ago with no evidence of recurrence. The patient did not have personal or familiar history of cutaneous malignancy.

**Figure 1:**
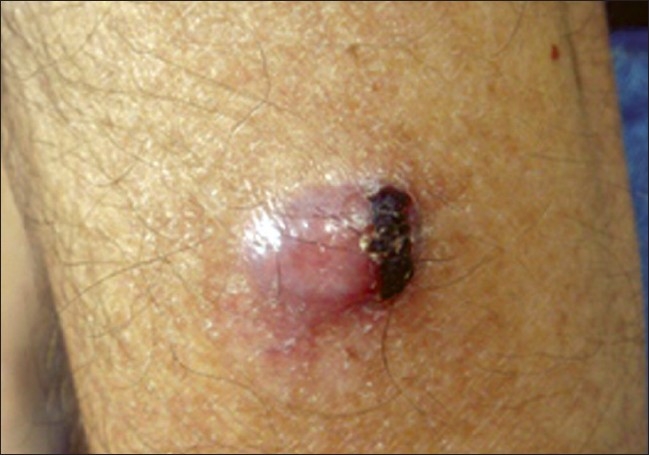
On the right arm, an erythematous, dome-shaped, nodular lesion, 1.3 cm in diameter with a serohemorragic crust on its surface could be observed. No other relevant lesions were found on total skin examination

Physical examination revealed an erythematous, dome-shaped, nodular lesion, 1.3 cm in diameter, firm to palpation and movable with a serohemorragic crust on its surface, located on the right arm [Figure 1]. Clinical examination did not reveal other relevant cutaneous lesions.

**Figure 2:**
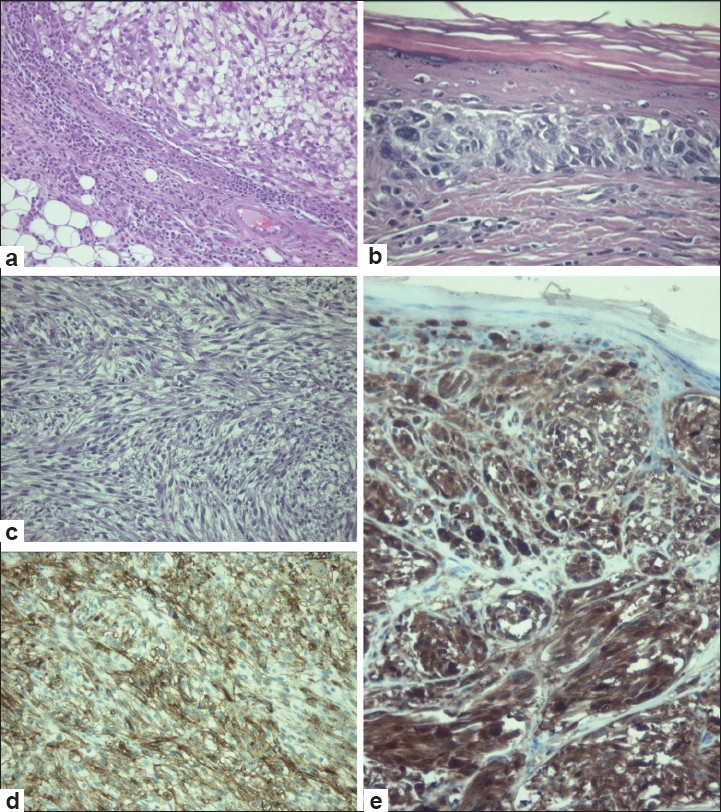
(a) In a low power picture, oval to polygonal in shape cells with a clear cytoplasm and irregular nuclear contours and complex nucleoli can be observed. Cells were disposed in nests separated by fibrous connective tissue. (b) Intraepidermal involvement can be observed. (H&E, ×40); (c) In dermis, cells were disposed in nests separated by fibrous connective tissue, showing an alveolar pattern (H&E, ×40); (d) Neoplastic cells displayed positivity for HMB 45; and (e) Positivity for S-100 protein.

**Figure 3a and b:**
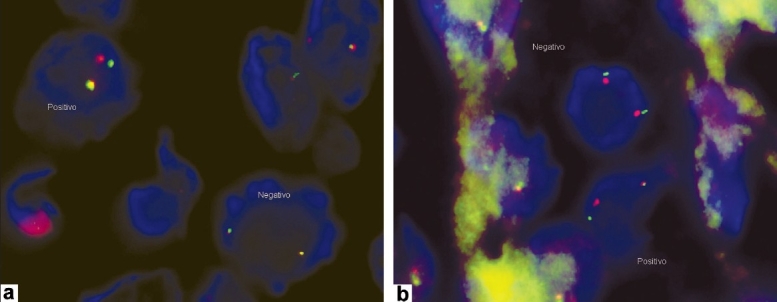
FISH technique: A single-stranded probe was used to hybridize with a specific DNA sequence of the sample. In this case, we used the LSI CHOP (12q13) Dual Color, Break Apart Rearrangement Probe (Abbott Laboratories), which showed the nuclei of normal cells with two fusion signals, whereas the signal pattern of abnormal cells was one orange, one green, and one fusion signal

Skin biopsy was performed. Histologically, the cutaneous specimen demonstrated epidermal involvement similar to MM. Predominantly, the tumor was composed of oval to polygonal cells with clear cytoplasms and enlarged, irregular, hyperchromatic nuclei with nucleoli. Tumoral cells were arranged in sheets or small nests [Figure 2a]. Junctional activity could be observed in the tumor, with nests of proliferating melanocytic cells showing cytologic atypia in the basal layer. At the lateral margins, atypical melanocytes nested in dermoepidermal junction [Figure 2b], showing features of a remnant nevus in the specimen. In dermis, cells were disposed in nests separated by fibrous connective tissue, showing an alveolar pattern [Figure 2c]. The depth of invasion was 11mm. Melanin granules were not detected. Mononuclear inflammatory infiltrate, necrotic, and hemorragic areas were also found. Superficial ulceration was observed. Neoplastic cells displayed positivity for HMB-45 and S-100 protein. Immunohistochemistry also showed intraepidermal positivity [Figure 2d and e]. Stains for CEA and cytokeratin were negative. Translocation t(12; 22)(q13; q12) was found in cytogenetic analysis by fluorescence in situ hybridization (FISH). FISH technique was performed using the LSI CHOP (12q13) Dual Color, Break Apart Rearrangement Probe (Abbott Laboratories). Our results showed 20–30% of rearrangement in Chop gene over the analyzed tissue [Figure 3a and b].

The intraepidermal involvement observed in the histologic study resembled a MM. Nevertheless, the histopathologic, cytogenetic and immunohistochemical profile of this neoplasm was diagnostic of invasive CCS. There was no other identifiable primary cutaneous neoplasm that would give rise to the metastatic deposit. Finally a diagnosis of primary CCS was made based on the strength of the genetic studies.

Axillary lymph node and lung metastases were observed on magnetic resonance imaging. A lung biopsy confirmed the diagnosis of metastatic CCS. Radical surgery, including regional lymph node dissection was performed and adjuvant radiotherapy was instaured.

## Discussion

CCS is a recently described variant of sarcoma characterized by prominent clear cell features similar to clear cell melanoma.[7] Primary CCS usually arises in deeper soft tissues, in association with fascia, tendons, or aponeuroses. Histopathologic criteria that support the classification of CCS as a separate entity include the presence of spindle and clear cells, absence of nuclear atypia, and small and inconspicuous nucleoli. Clinically, CCS is most often present in young adults, with a slight female predominance, as a slow-growing but often painful nodule on the extremities.[4–6] Most cases occur on the lower limbs, with feet predilection. Upper limb is affected in about 25% of cases.[4,5] Upon incomplete excision, local recurrence and metastasis (lymph nodes, lungs or bones) may occur.[3,4]

It has been suggested that CCS may be conclusively diagnosed using cytology, immunohistochemistry (HMB- 45+ and S-100+ stains), cytogenetic analysis (demonstrating the specific translocation), and electron microscopy (to supply ultrastructural evidence for the presence of melanosomes).[2,8–10] However, CCS shares a similar immunohistochemical profile with MM, with two exceptions: (i) CD68 is more specific for MM and has not been reported positive in CCS, and (ii) CCS displays occasional positivity for chromogranin while MM does not [Table 1].[3,11]

**Table 1:** Immunohistochemical characteristics for differential diagnosis

Marker	Malignant melanoma	Clear cell sarcoma	MPNST	Follicular dendritic tumor	Interdigitating cell tumor	PEC-omas
S-100	+++	+++	++	+/−	++	+++
HMB-45	++	++	−	−	−	++
Melan A	+++	++	−	−	-	++
Tyrosinase	+++	++	−	−	−	++
Chromogranin	−	+/−	−	−	−	+
CD68	+/−	−	−	−	++	+
Desmin	−	−	−	−	−	++
Vimentin	+++	+++	+++	+	+	+
EMA	+/−	−	+/−	−	−	−
SMA	−	−	−	−	−	++

Many sarcomas are known to have translocations and gene fusions of great value in specific diagnosis. Characteristic translocation t (12; 22) (q13; q12), has been considered pathognomonic for CCS.[12] This translocation has been identified in 70-90% of CCS cases using cytogenetic studies and reverse-transcriptase polymerase chain reaction.[13] Nevertheless, this cytogenetic rearragement is characteristic but not entirely unique for CCS, because similar fusion genes can also be found in angiomatoid fibrous histiocytoma.[14] In this case, we used FISH technique for cytogenetic studies. It is based on denaturalization and renaturalization of the DNA molecule, by using heat changes and also on the complementarity of base pairs. A single-stranded probe is used to hybridize with a specific DNA sequence of the sample.

To date, this translocation has not been identified in cutaneous MM. However, microsatellite instability (a variation in the lengths of short repeat DNA segments in the genome) and genetic alterations involving chromosomes 1, 5, and 6 have been implicated in MM pathogenesis, but are rare or absent in CCS.[15] Therefore, cytogenetic data could be a clue for establishing the correct diagnosis.[5–7,11]

It is important to be aware with the wide range of clinical and pathological differential diagnosis of clear and spindle-cell neoplasms. Most important differential diagnosis is MM, including metastatic MM with unknown primary site or rare variants as clear cell melanoma or balloon cell melanoma.[8–10]

Follicular dendritic and interdigitating cell tumors, MPNSTs, paraganglioma-like dermal melanocytic tumor, hidradenocarcinoma, perivascular epithelioid cell-omas (*PEC-omas*), clear cell basal cell carcinoma, clear cell syringoma, balloon cell nevus or melanoma, xanthoma, hibernoma, sebaceous neoplasm, atypical fibroxanthoma, or hypernephroma should also be considered. Their common embryologic origin, similar histologic, and immunohistochemical features make differential diagnosis difficult.[3–6,16–18]

Distinction of CCS from metastatic MM is important due to the different treatment and prognosis.[19] Rarity of CCS makes it difficult to draw conclusions regarding prognostic factors. Tumor size ≥5cm, presence of necrosis and perhaps, DNA-index have been found as poor prognosis factors.[1,20,21] Regional lymph nodes or lung metastases have been reported in one third of the patients.[3,4] Five-year survival rates have been estimated to range from 48-67%.[3,11]

In conclusion, an interesting clinical case of CCS in an adult, arising in the upper limb has been reported. In addition, most important differential diagnoses have been reviewed. The intraepidermal involvement over this tumor (a finding that resembles MM), in a tumor that morphologically resembles MM, in a site that is more typical of melanoma make this final diagnosis difficult. To our knowledge, this is the first reported case of CCS with intraepidermal involvement. In our patient, definitive diagnosis was only possible based on the strength of the genetic studies. Differentiation from the clear or spindle-cell neoplasms, metastatic MM, and unusual MM subtypes is an essential component in patient management. Pathologist and clinicians need to be aware of the aforementioned entities, so that an early diagnosis and treatment may improve the prognosis.
